# Supplementary figures and images for: Human cytomegalovirus IE2 drives transcription initiation from a select subset of late infection viral promoters by host RNA polymerase II
Source: PLoS Pathog. 2020 Apr 6;16(4):e1008402. doi: 10.1371/journal.ppat.1008402 (PMC7162547; doi:10.1371/journal.ppat.1008402)

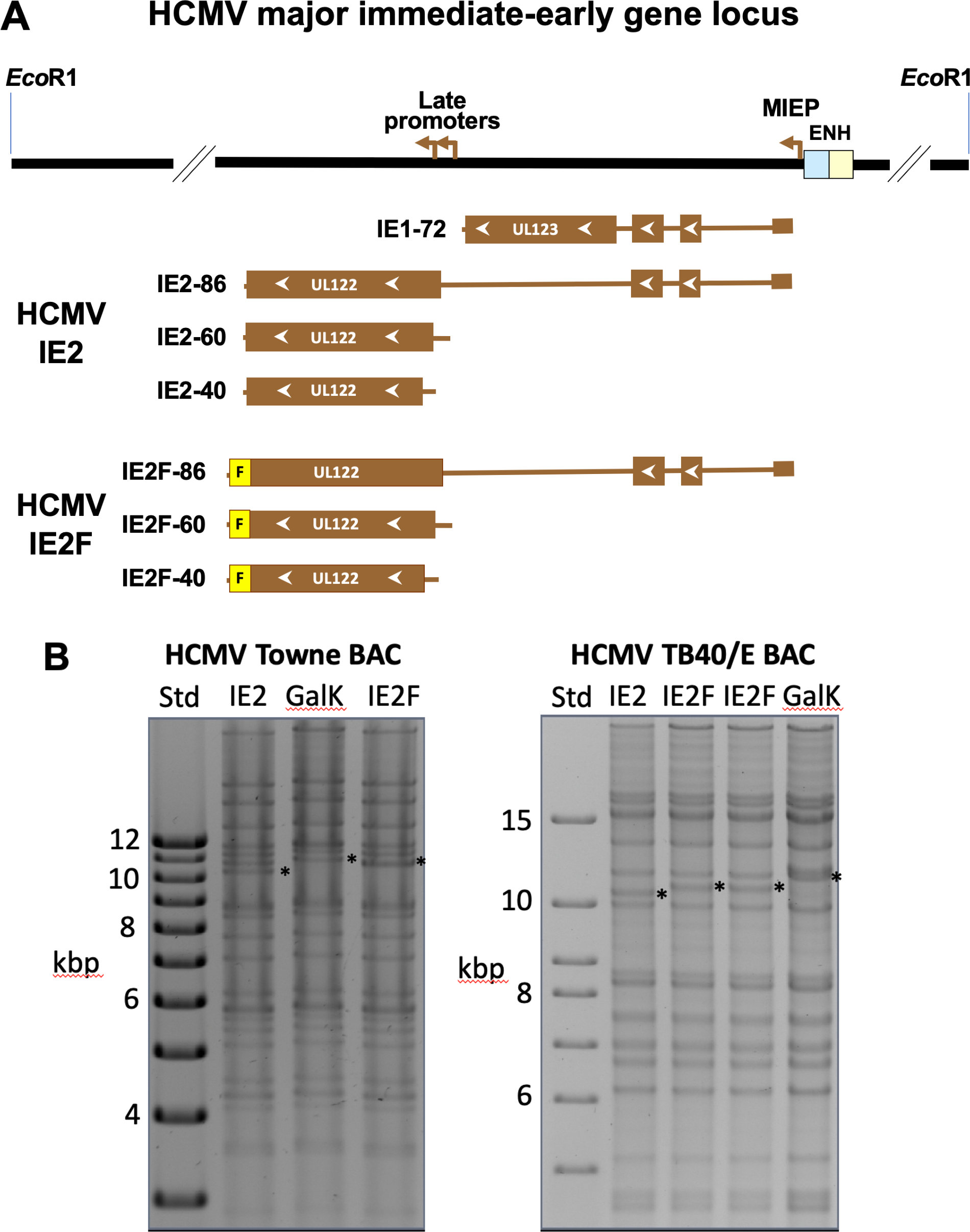

Supplement: S1 Fig — (A) Map of the HCMV MIE gene locus in relation to EcoRI sites and the IE2 proteins expressed from this locus in late infection. The HCMV IE2F BAC construct has FKBP12F36V (F) fused in-frame to the carboxyl ends of IE2 family members, whereas HCMV IE2 BAC has wildtype IE2 proteins. (B) HCMV Towne and TB40/E IE2 and IE2F BAC genomes digested with EcoRI and fractionated on 0.6% agarose gels. The GalK BACs were derived from IE2 BACs and serve as intermediates for creating the IE2F BACs. Two TB40/E IE2F BACs were derived from separate recombination procedures. The EcoRI fragments for IE2 and IE2F are ~10.1 kbp and ~10.4 kbp, respectively. Asterisks mark positions of these EcoRI fragments. (TIF) [file ppat.1008402.s001.tif]

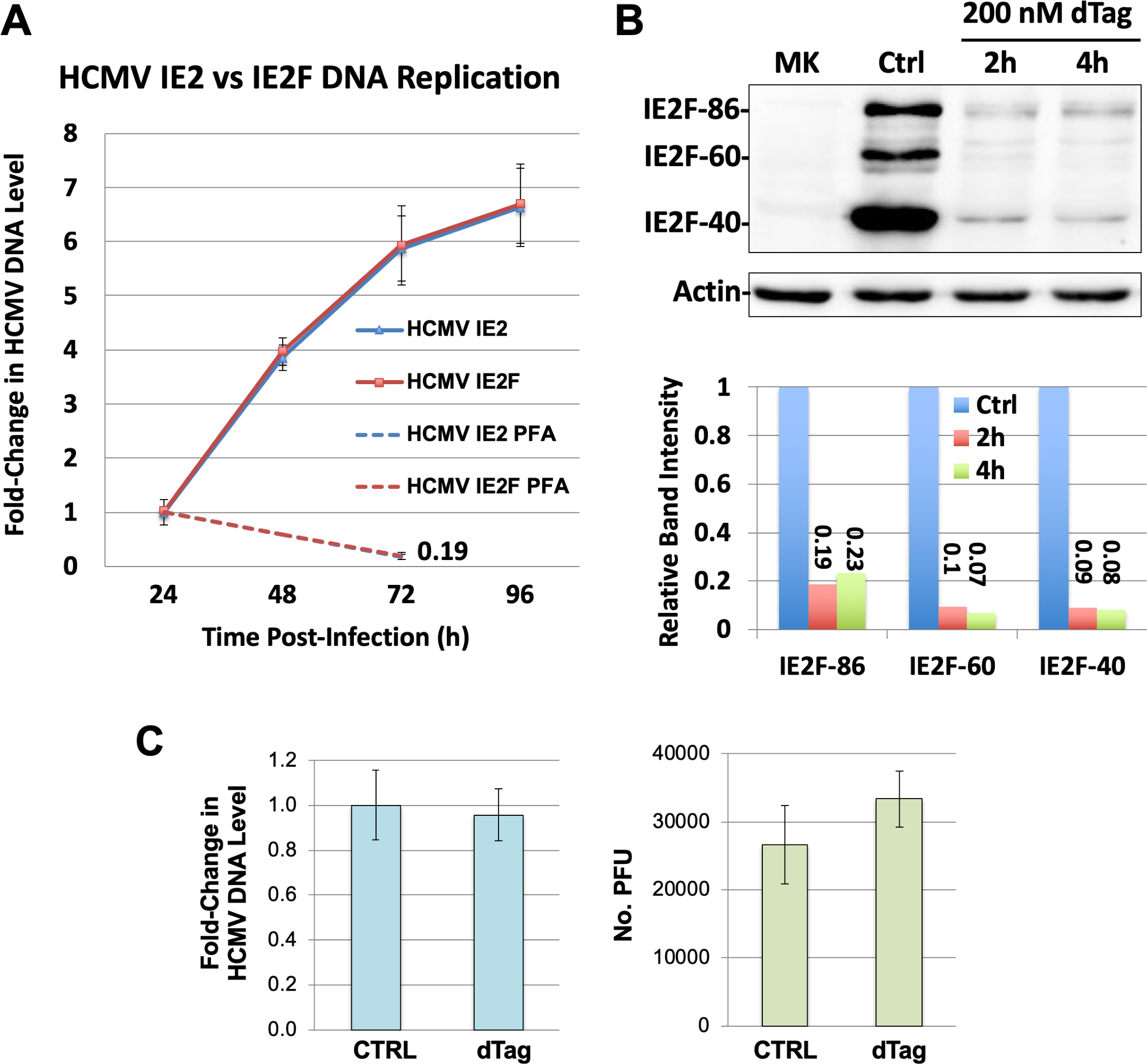

Supplement: S2 Fig — (A) At 72 h pi in HFF, the amounts of genomes for both HCMV Towne IE2 and IE2F viruses (MOI of 3) increased approximately 31-fold, compared to amounts of viral genomes exposed to the PFA inhibitor (200 μM) of viral DNA synthesis throughout the infection. HCMV DNA in triplicate infections was quantified by real-time PCR and normalized to host GAPDH. (B) HFF infected with HCMV Towne IE2F at MOI of 3 were treated with vehicle control (CTRL) or 200 nM dTag at 90–96 h pi. Western blot performed at 96 h pi and band intensities of IE2F-86, IE2F-60, and IE2F-40 determined for dTag relative to CTRL. Mock, MK. (C) Effect of 6-h dTag (200 nM) versus CTRL treatment at 90–96 h pi on production of HCMV DNA and cell-associated infectious progeny (MOI 0.5) at 96 h pi. Results of 3 biological replicates. HCMV DNA quantified as in panel A. PFU determined by viral plaque assay on HFF. (TIF) [file ppat.1008402.s002.tif]

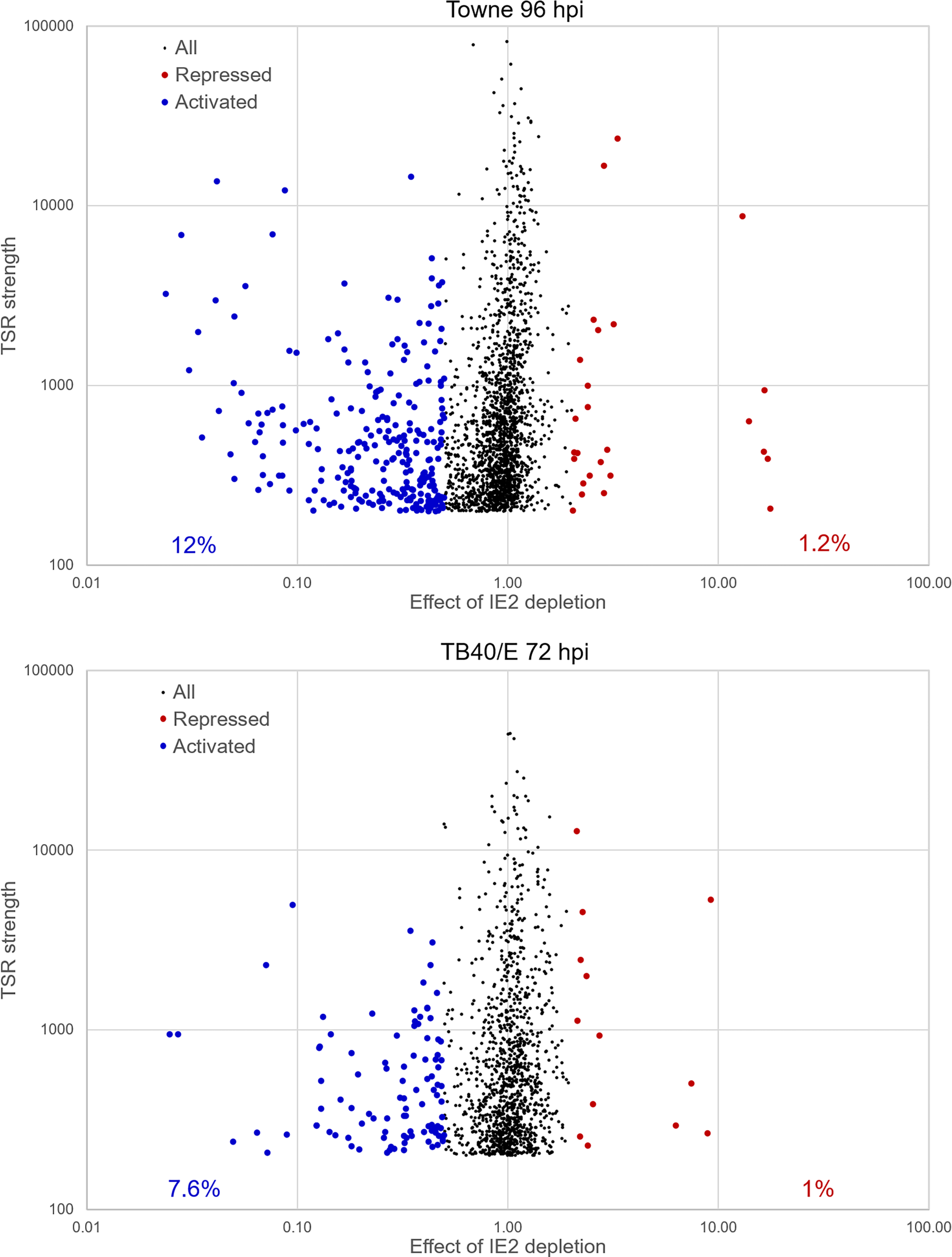

Supplement: S3 Fig — TSR strength determined for all viral TSRs. Repressed and activated TSRs increase and decrease in strength, respectively, when IE2F is depleted. Repressed TSRs containing a dTag / CTRL ratio greater than 2 are red. Activated TSRs with the ratio less than 0.5 are blue. TSRs not changing by this degree are indicated by a small black dot. (TIF) [file ppat.1008402.s003.tif]

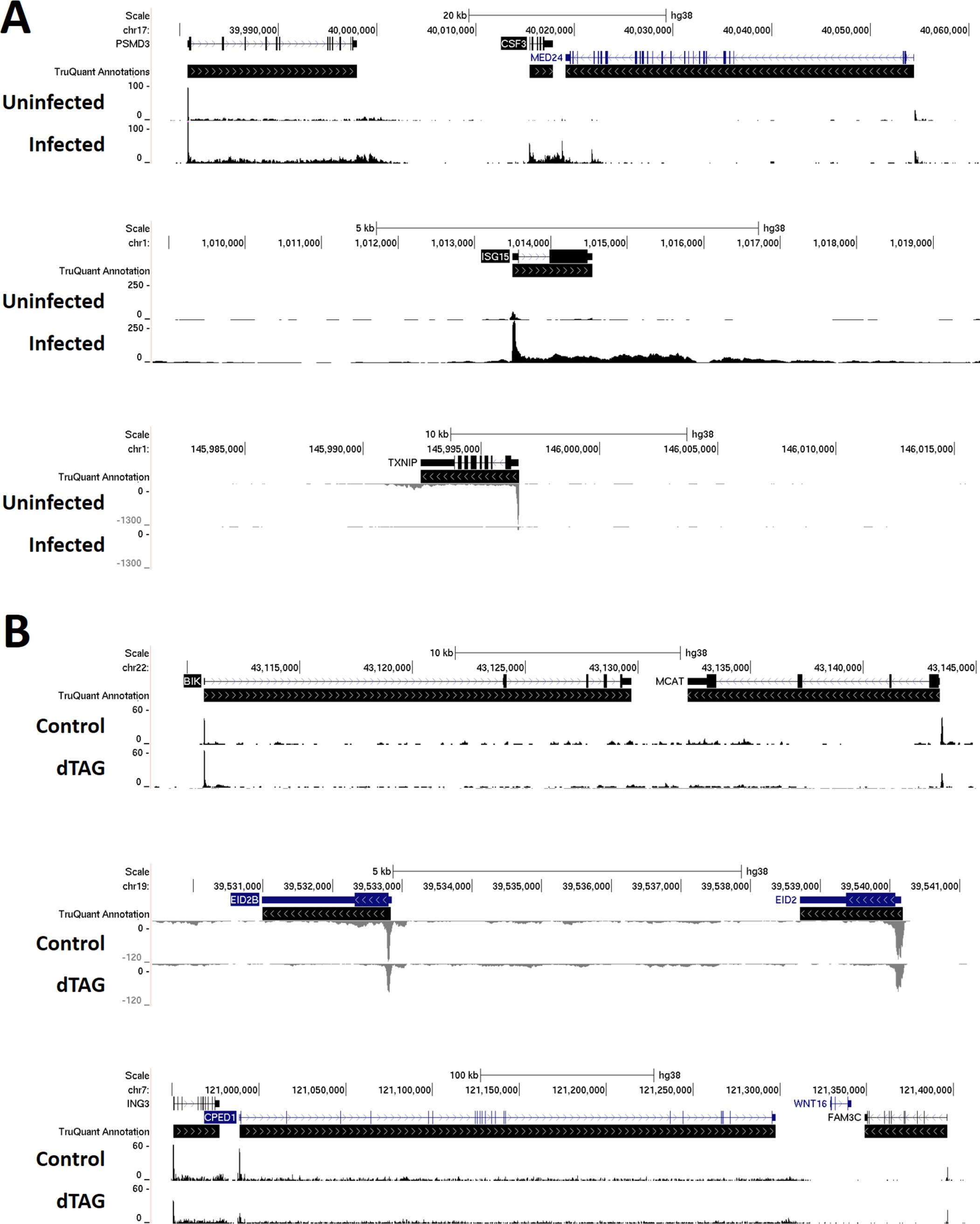

Supplement: S4 Fig — (A) UCSC Genome Browser views of normalized PRO-Seq data for highly induced (CSF3, ISG15) and inhibited (TXNIP) genes upon HCMV TB40/E infection. GENCODE v31 basic annotations are displayed in black. TruQuant annotations are shown as black rectangles underneath GENCODE v31 annotations. (B) PRO-Seq data for largest changes upon IE2F depletion. BIK was selected as one of the most upregulated genes upon IE2 depletion. EID2B and CPED1 were identified as the most downregulated genes. (TIF) [file ppat.1008402.s004.tif]

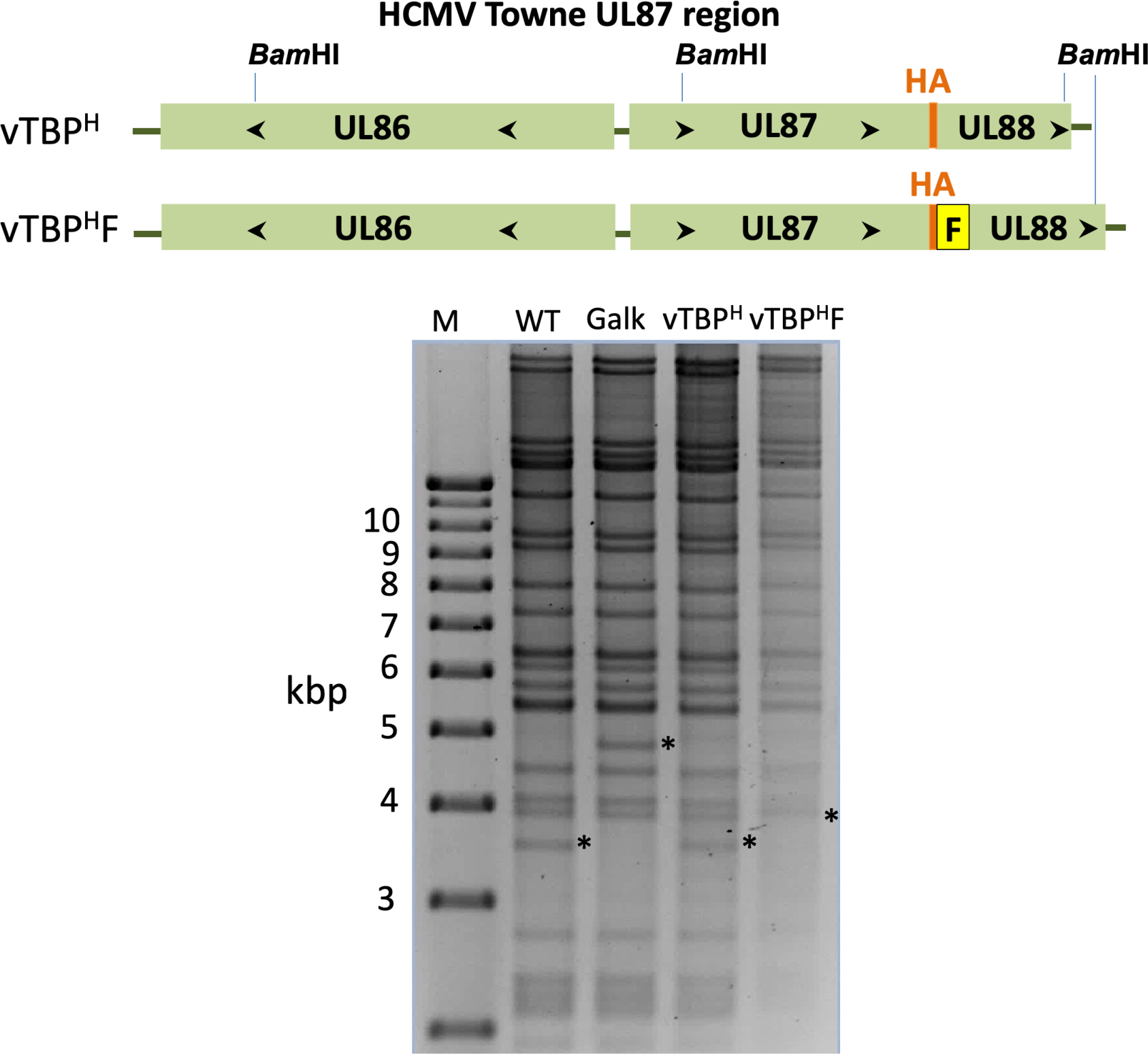

Supplement: S5 Fig — (A) Map of HCMV Towne UL87 gene with carboxyl end fused to HA alone or HA plus FKBP12M (F). Positions of BamHI sites depicted. (B) Agarose gel electrophoresis. BamHI digestion of HCMV WT Towne BAC and recombinants GalK (placed at the C-terminus of UL87), vTBPH, and vTBPHF yields fragments of 3.48, 4.7, 3.5, and 3.8 kbp, respectively. (TIF) [file ppat.1008402.s005.tif]

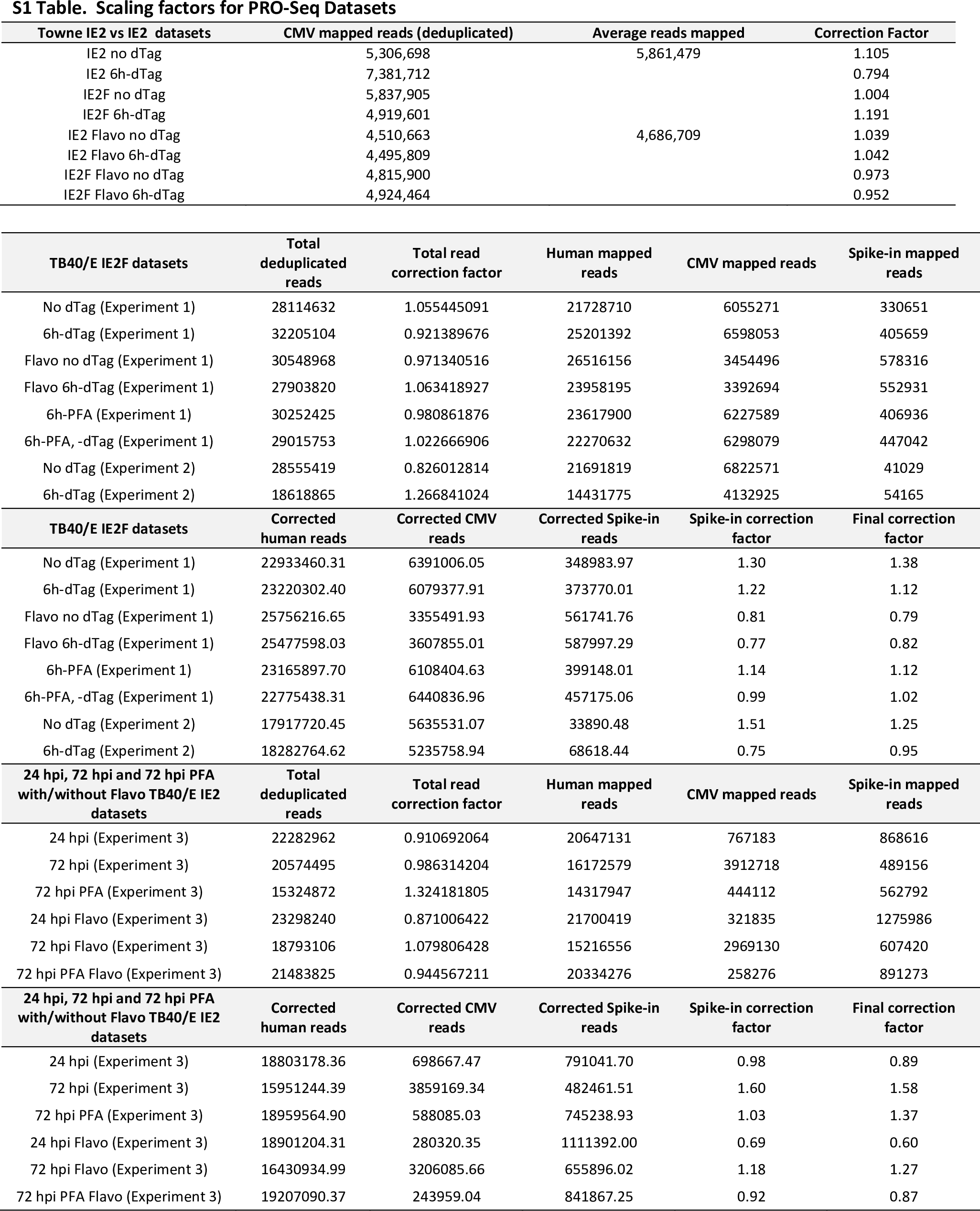

Supplement: S1 Table — (TIF) [file ppat.1008402.s006.tif]

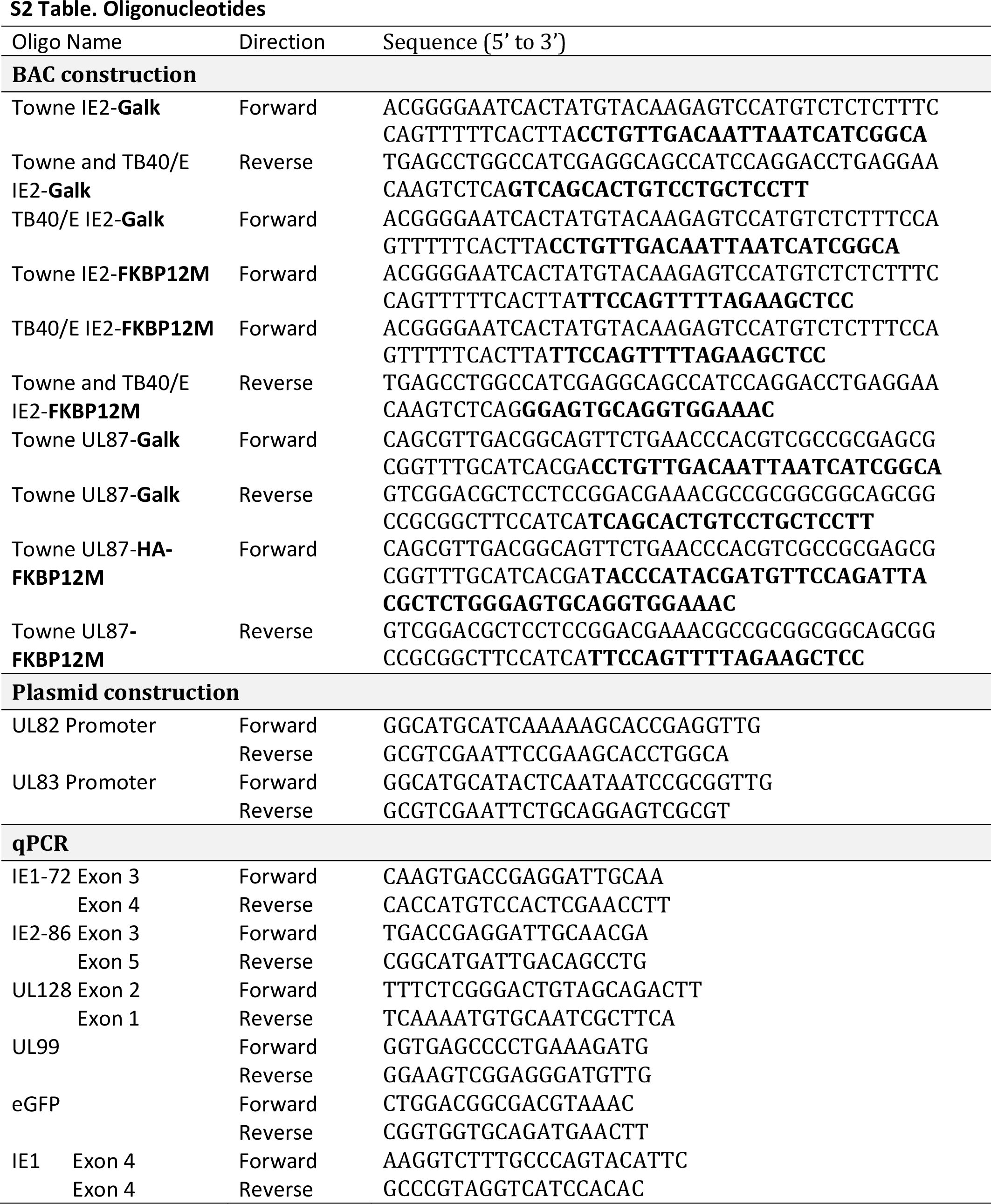

Supplement: S2 Table — (TIF) [file ppat.1008402.s007.tif]
