# Supplementary material for: Human cytomegalovirus IE2 drives transcription initiation from a select subset of late infection viral promoters by host RNA polymerase II
Source: PLoS Pathog. 2020 Apr 6;16(4):e1008402. doi: 10.1371/journal.ppat.1008402 (PMC7162547; doi:10.1371/journal.ppat.1008402)
Supplement: Attachment — Submitted filename: Responses to Reviewers.pdf [file ppat.1008402.s010.pdf]

UNIVERSITY OF IOWA HEALTH CARE

Roy J. and Lucille A. Carver
College of Medicine
University of Iowa
Department of Internal Medicine
Division of Infectious Diseases
200 Hawkins Dr., SW34 GH
Iowa City, Iowa 52242-1009
319-356-7055 Phone
319-384-7208 ***Fax***

January 24, 2020

Dear PLOS Pathogens Editors,

Thank you for serving as the Editors for PLOS Pathogens. We are grateful to the reviewers for their advice on ways to improve the manuscript. We have changed the manuscript to incorporate this advice and describe below how we have addressed the issues raised by reviewers.

Reviewer 1:

1. ***Need to address if IE2 is activating early-late and/or late viral promoters***.
   We thank Reviewers 1 and 3 for prompting us to do a more in-depth analysis of the PFA effect on IE2-activated promoters. The revised manuscript has results of a comparative quantitative bioinformatic analysis of transcription start regions (TSRs) across the viral genome. We developed a PFA-sensitivity index (PSI) (vertical axis in **new Figure 4**) that compares the relative expression of each TSR at 72 h post-infection with and without PFA to provide information about "lateness" of each TSR. The horizontal axis in new Figure 4 shows the quantitative effects of IE2 depletion for all TSRs, indicating which viral TSRs are activated or repressed by IE2. The plot also shows which TSRs have a TATT positioned at -37 to -30 upstream of the maximum transcription start site (TSS). This puts all viral TSRs/TSSs into quantitative perspective in relation to lateness and IE2 effect. **Revised Table 1** further puts this new information in context. **New Fig. S3** reveals that the quantitative effects of IE2 depletion are similar for Towne and TB40/E strains. We've also **added a supplementary xl spreadsheet** containing all the TSR data and computations (as suggested by Reviewer 1). For the PSI analysis, we used the datasets for 72 h pi (+ or – PFA) PRO-Seq plus Flavo because Flavo datasets more accurately represent TSRs. These datasets had been generated in parallel with those used in the original manuscript, but we had not included them as a matter of discussion. These **datasets and browser tracks have been added** to the revised manuscript. We have also added the matching Flavo dataset and browser track for the 24 h pi PRO-Seq shown in Figure 2 to provide more information about TSR activity prior to viral DNA replication. All datasets have been uploaded to GEO and browser tracks are viewable via the Github link in the manuscript. The discussion section was revised to include the new information and minor changes have been made in the title and abstract.
2. ***Information about the relative roles of the IE2 isoforms unnecessary because this topic is not the focus of this study***.
   We opted to keep the information in the introduction about the function of the individual IE2 isoforms to point out that the 3 different IE2 isoforms may have overlapping function, as this provided rationale for depleting all 3 isoforms to get at IE2's effect on transcription in late infection. We have deleted the 2 sentences in the discussion that speculates on the individual roles of these proteins because this side issue (as pointed out by the reviewer) was not adequately investigated.

Reviewer 2:

1. ***Mechanism of late gene expression is not adequately explored beyond the application of PRO-Seq and inspection of promoter elements.***

We are pleased that the reviewer finds merit in extending this line of investigation. How IE2 drives transcription initiation at a select subset of viral early-late and late transcription start sites in late infection (while not affecting host transcription) is an area of ongoing research.

Additional comments:

1. ***Chromatin was not examined, so the following statement in the discussion section is not appropriate "Our results support a model in which replicated HCMV genomes rid of repressive chromatin allow IE2 and LTFs to carry out…".***
   We agree that our word choices should have been better. We have changed this sentence to state that "Our results are consistent with the hypothesis that at least a fraction of replicated HCMV genomes are rid of repressive chromatin to allow IE2 and LTFs to activate the viral late infection promoters."
2. ***Model figure in discussion section is vague.***
   We agree that the figure is unnecessary. It has been deleted, which makes space for another figure of results that requires additional text to report and discuss.
3. ***Talk about host PIC components but not data.***
   The sentence in discussion pertaining to host PIC components were edited to clarify. "This outcome is possibly the result of a strengthening of preinitiation complex assembly or loading onto the promoters." We removed remaining sentence mentioning host GTFs.
4. ***Questions about the dTag approach.***
   A. ***How well does this approximate the IE2 null situation?*** The inability to produce cell lines that express IE2 for trans-complementation studies has stymied progress in this area for nearly 30 years. Debbie Spector has come the closest to developing a system to do this (JVI 2007), but it has important limitations, has not been used by others, and does not involve expression of all the IE2 isoforms. We have added a sentence to the discussion to acknowledge the limitation of our method. "Because the methodologic approach applied in these studies does not eliminate 100% of existing IE2 proteins over a 6-h timeframe, our list of viral promoters regulated by IE2 in late infection may be incomplete. The quantitative effects of IE2 are also likely to be greater than what is shown due to some remaining IE2"
   B. ***Does dTag affect the viral lifecycle?***
   For PRO-Seq studies, dTag was added for the last 6 h of a 72 or 96 h infection. In this short timeframe, the drug effect has negligible effect on viral DNA replication or amount of infectious virus in cells. These results were added to panel C in supplementary figure, **Fig. S2C**.
   C. ***Change in HCMV DNA amount with replication is smaller than expected (Supplemental Fig. S2A).*** At MOI of 3, a considerable amount of replication-defective viral particles is part of the cell-associated HCMV DNA at 24 h pi. The hatched line in Fig. S2A showed 80% decline in viral DNA amount at 72 h pi in cells maintained in the presence of the PFA inhibitor of viral DNA synthesis. We have amended the **Fig. S2A legend** by adding the sentence - "At 72 h pi in HFF, the amounts of genomes for both HCMV Towne IE2 and IE2F viruses (MOI of 3) increased approximately 32-fold, compared to amounts of viral genomes exposed to the PFA inhibitor of viral DNA synthesis throughout the infection."
5. ***Omission of Fig. S3.***
   We apologize for the missing figure. We have included a **new Fig. S3** that is paired with new Fig. 4. There are a total 5 supplementary figures.

Reviewer 3:

1. ***Need to more thoroughly evaluate the effects of PFA on transcription.*** This is addressed above.
2. ***vTBP's role on viral transcription not evaluated by PRO-Seq.*** We agree that this is an important area of investigation and will tell us if IE2 is acting on a few of the many viral promoters activated by LTFs. However, to rigorously carry this out will require considerable expense to generate duplicate datasets with appropriate controls. We also anticipate that the results will take us in a direction that would distract from the IE2 focus in this manuscript. Therefore, we have opted against including this line of investigation in the current report.
3. ***Need more comprehensive evaluation of transcription start regions that have an upstream TATT that are not affected versus affected by IE2***. Thank you for the advice. We have performed the bioinformatic analysis and integrated this information with the results of the PFA effect on viral transcription. The results are displayed in a **new Fig. 4** and are reported and discussed in the revised manuscript. The results are interesting and worthy of further investigation. As we describe in the revised manuscript, the PSI lateness scores are very similar for IE2-activated and -unaffected viral TSRs having TATT suitably positioned upstream of the TSS.
4. **Sections of discussion are unclear.** The sentences mentioned as unclear were either deleted or rewritten.

Thank you again for the time and care you have taken in helping make this a better publication

Sincerely,

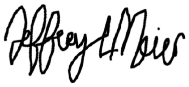

Jeffery L. Meier, MD
Department of Internal Medicine
University of Iowa
jeffery-meier@uiowa.edu
